# Atrial myocyte-derived exosomal microRNA contributes to atrial fibrosis in atrial fibrillation

**DOI:** 10.1186/s12967-022-03617-y

**Published:** 2022-09-05

**Authors:** Hongting Hao, Sen Yan, Xinbo Zhao, Xuejie Han, Ning Fang, Yun Zhang, Chenguang Dai, Wenpeng Li, Hui Yu, Yunlong Gao, Dingyu Wang, Qiang Gao, Yu Duan, Yue Yuan, Yue Li

**Affiliations:** 1 Department of Cardiology, the First Affiliated Hospital, Harbin Medical University, Youzheng Street 23#, Nangang District, Harbin, 150001 Heilongjiang China; 2 NHC Key Laboratory of Cell Translation, Harbin Medical University, Harbin, 150001 Heilongjiang China; 3 Key Laboratory of Hepatosplenic Surgery, Ministry of Education, Harbin Medical University, Harbin, 150001 China; 4 Key Laboratory of Cardiac Diseases and Heart Failure, Harbin Medical University, Harbin, 150001 China; 5 Heilongjiang Key Laboratory for Metabolic Disorder & Cancer Related Cardiovascular Diseases, Harbin, 150081 China; 6 Institute of Metabolic Disease, Heilongjiang Academy of Medical Science, Harbin, China

**Keywords:** Atrial fibrillation, Atrial fibrosis, Exosomes, miR-210-3p, GPD1L

## Abstract

**Background:**

Atrial fibrosis plays a critical role in the development of atrial fibrillation (AF). Exosomes are a promising cell-free therapeutic approach for the treatment of AF. The purposes of this study were to explore the mechanisms by which exosomes derived from atrial myocytes regulate atrial remodeling and to determine whether their manipulation facilitates the therapeutic modulation of potential fibrotic abnormalities during AF.

**Methods:**

We isolated exosomes from atrial myocytes and patient serum, and microRNA (miRNA) sequencing was used to analyze exosomal miRNAs in exosomes derived from atrial myocytes and patient serum. mRNA sequencing and bioinformatics analyses corroborated the key genes that were direct targets of miR-210-3p.

**Results:**

The miRNA sequencing analysis identified that miR-210-3p expression was significantly increased in exosomes from tachypacing atrial myocytes and serum from patients with AF. In vitro, the miR-210-3p inhibitor reversed tachypacing-induced proliferation and collagen synthesis in atrial fibroblasts. Accordingly, miR-210-3p knock out (KO) reduced the incidence of AF and ameliorated atrial fibrosis induced by Ang II. The mRNA sequencing analysis and dual-luciferase reporter assay showed that glycerol-3-phosphate dehydrogenase 1-like (GPD1L) is a potential target gene of miR-210-3p. The functional analysis suggested that GPD1L regulated atrial fibrosis via the PI3K/AKT signaling pathway. In addition, silencing GPD1L in atrial fibroblasts induced cell proliferation, and these effects were reversed by a PI3K inhibitor (LY294002).

**Conclusions:**

Atrial myocyte-derived exosomal miR-210-3p promoted cell proliferation and collagen synthesis by inhibiting GPD1L in atrial fibroblasts. Preventing pathological crosstalk between atrial myocytes and fibroblasts may be a novel target to ameliorate atrial fibrosis in patients with AF.

**Graphical Abstract:**

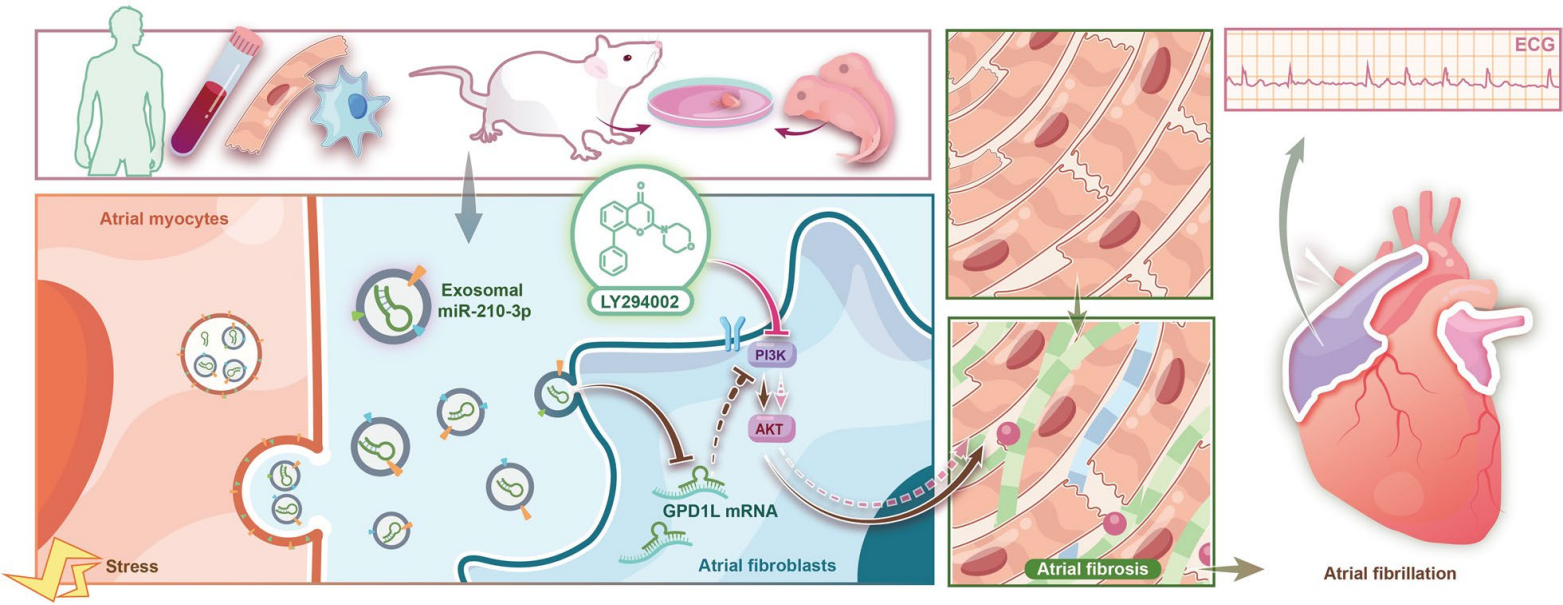

**Supplementary Information:**

The online version contains supplementary material available at 10.1186/s12967-022-03617-y.

## Introduction

Atrial fibrillation (AF) is the most common arrhythmia observed in clinical practice and independently increases the risks of mortality and morbidity due to stroke, heart failure, and impaired quality of life, resulting in a substantial public health burden [1]. Cardiac fibrosis is an important contributor to the development of various cardiovascular diseases, including AF. Cardiac fibrosis is characterized by an abnormal balance of collagen deposits, such as collagen I and collagen III[2]. During the past few decades, several treatment strategies have been developed; however, the exact molecular mechanisms and efficient therapeutic approaches underlying AF-associated atrial fibrosis remain unclear.

An increasing number of studies highlight that extracellular vesicles (EVs) mediate cellular communication by delivering molecules in different pathological processes, including inflammation, fibrosis and angiogenesis [3]. EVs are composed of a lipid bilayer containing transmembrane proteins and encapsulated cytosolic proteins and RNAs. Different types of EVs are secreted and have been classified according to their subcellular origin [4]. Other types of vesicles, such as exosomes, are generated in multivesicular bodies (MVBs) and are secreted when these MVBs fuse with the plasma membrane [5]. Exosomes are small extracellular membrane vesicles of endocytic origin that are released by fusion with the cell membrane; their diameters range from 30 to 200 nm [6]. Exosomes are capable of carrying diverse molecules, such as proteins, lipids, and microRNAs (miRNAs), to mediate complex intercellular communication [7]. Exosomal miRNAs contribute to the progression of cardiac hypertrophy [8]. Several studies have shown that cardiomyocyte-secreted exosomal miRNAs promote the proliferation and differentiation of cardiac fibroblasts [9].

Previous studies have shown the underlying functions of miRNAs that are expressed at high levels in patients with different clinical types of AF, such as miR-99a-5p, miR-214-3p and miR-342-5p [10–13]. In addition, miR-150 expression is also altered and correlated with AF development, and circulating miR-150 levels are lower in patients with AF than in patients in sinus rhythm (SR) [14]. Notably, miR-210-3p reduces the formation of aortic atherosclerotic lesions and inhibits lipid deposition and inflammation in plaques but increases collagen aggregation to promote plate stability in mice [15].

In the present study, we determined whether exosomal miR-210-3p derived from atrial myocytes is specifically associated with the proliferation of atrial fibroblasts. Targeting miR-210-3p-mediated pathological communication between atrial fibroblasts and atrial myocytes may be a novel strategy to treat fibrosis during AF progression.

## Methods

### Human experiments

Six patients were recruited from the Department of Cardiology and the inpatient ward at The First Affiliated Hospital of Harbin Medical University from June 2020 to June 2021 (Ethical approval number: IRB-AF/SC-04/02.0) (Harbin, Heilongjiang, China). Serum samples from 3 patients with SR and 3 patients with AF were used to extract exosomes for the miRNA sequencing analysis. Then, one hundred patients were recruited for the analysis of miR-210-3p levels in plasma exosomes, including 50 patients with SR and 50 patients with AF Serum exosomes were extracted using an exosome kit (EXOQ5A-1, USA), and miRNAs were extracted from exosomes. Exclusion criteria included patients with infectious diseases, severe liver and kidney conditions, malignant tumors and severe cardiac hypofunction.

### Animal experiments

Male SD (Sprague–Dawley) rats and miRNA-210-3p knockout (KO) rats aged 6–8 weeks (weighing 200–300 g) were purchased from Beijing Laboratory Animal Center (Beijing, China). The experimental procedures were approved by the Institutional Animal Ethical Committee of The First Affiliated Hospital of Harbin Medical University. The experiments were performed according to NIH Guidelines for Care and Use of Laboratory Animals. The rats were maintained in individually ventilated cages (at 22 °C, 12 h light/dark cycle) with free access to standard laboratory chow (Ethical approval number: 2019044).

### Treatment of rats with AgomiR-210-3p

SD rats were injected with normal saline, NC (negative control) reagent and miR-210-3p agonist (50 nmol) through the tail vein every  days to overexpress miR-210-3p in vivo. After 4 weeks, the rats were anesthetized with 10% chloral hydrate (0.3 mg/kg), and the hearts were removed. The AgomiR-210-3p and control sequences were purchased from RiboBio, Guangzhou, China.

### Procedures used for Ang II treatment in rats

Then, the rats were randomly divided into four groups (7 rats per group): the WT group, WT + Ang II group, KO-miR-210-3p group and KO-miR-210-3p + Ang II group. Ang II (500 ng/kg/min, Sigma-Aldrich) was dissolved in 200 μl of sterile saline and loaded into a mini-osmotic pump (ALZET 2004, USA). For pump insertion, rats were anesthetized with 10% chloral hydrate (0.3 mg/kg), and the upper back was cleaned with 70% ethanol. A 1.0 cm skin incision was made in the upper back, and then a mini-osmotic pump was implanted under the skin. In the control group, 200 μl of saline were added to the mini-osmotic pump. Four weeks later, the rats were anesthetized, and cardiac tissues were removed, fixed with 10% neutral formalin and preserved at − 80 °C until further analysis.

### Electrophysiological studies

Electrophysiological studies were performed after 4 weeks to evaluate the effects of the vehicle and Ang II on the atrium. A distal quadripolar pacing electrode (Medtronic Inc., Minneapolis, MN, USA) was firmly attached to the free wall of the left atrial appendage. The atrial effective refractory period (AERP) was measured at a basic cycle length of 100 ms with a train of 8 basic stimuli (S1), followed by a premature extra stimulus (S2). The S1-S2 intervals were decreased in 5 ms steps until S2 failed to produce the atrial response, then increased by 10 ms, and finally decreased in 2 ms steps until S2 failed to capture. The longest S1-S2 interval that failed to capture was defined as the AERP. The AERP was recorded three times, and then we obtained the mean value for the three AERPs. The induction rate of AF was tested by burst pacing 10 times. AF was defined as a rapid, irregular atrial rhythm with a duration longer than 1000 ms. The AF incidence was defined as the percentage of successful inductions of AF.

### Echocardiographic measurements

Transthoracic echocardiography was performed on rats at baseline Day 0 and Day 28 after treatment to evaluate the structure and function of the atrium and ventricle. Rats were anesthetized with 10% chloral hydrate (0.3 mg/kg) and placed on a table in the left lateral decubitus position, after which two-dimensional images and M-mode tracings were recorded. Echocardiographic measurements included the left atrial diameter (LAD), right atrial diameter (RAD), interventricular septal thickness (IVST), left ventricular end-diastolic dimension (LVEDD), left ventricular end-systolic dimension (LVESD), left ventricular ejection fraction (LVEF), and left ventricular shortening rate (LVFS).

### Histological analysis

Hematoxylin and eosin staining and Masson’s trichrome staining were performed. The left atrial tissue was fixed with 10% phosphate-buffered formalin, embedded in paraffin, sliced into 4 μm serial sections, and subjected to a pathological examination following hematoxylin and eosin staining and Masson's trichrome staining. Masson's trichrome staining was performed to evaluate atrial fibrosis. The collagen fibers are stained blue, while the atrial myocytes are stained red. The semiquantitative analysis of the proportion of collagen fibers was conducted using Image-Pro Plus 6.0 software, and the results were reported as the ratio of fibrotic tissue to total tissue.

### Atrial fibroblasts and atrial myocytes isolation and culture

Atrial fibroblasts and atrial myocytes were isolated from 1-to 3-day-old SD rats. Hearts were minced and mixed with 0.25% trypsin. Cell suspensions were centrifuged and resuspended in Dulbecco’s modified Eagle’s medium (HyClone) containing 10% fetal bovine serum, 100 µg/ml penicillin and 100 μg/ml streptomycin under standard culture conditions (37 °C, 5% CO_2_). Atrial fibroblasts were isolated by removing atrial myocytes through the selective adhesion of nonmyocytes at a 1.5 h preplating interval. Atrial fibroblasts and atrial myocytes were treated when the cell confluence reached 70–80% and were used in our experiments.

### Exosome isolation and labeling

Exosomes were isolated from the atrial myocytes supernatant by ultracentrifugation. The cell culture supernatant was centrifuged at 300×*g* for 10 min, 2,000×*g* for 10 min, and 10,000×*g* for 30 min, followed by filtration through a 0.22 μm filter to eliminate cells, dead cells, and cellular debris. For exosomes purification, the supernatant was ultracentrifuged at 100,000×*g* for 70 min, followed by an additional washing step of the exosome pellet with PBS and centrifugation at 100,000×*g* for 70 min (Ultracentrifuge, Beckman Coulter, L8-70 M).

Exosomes were isolated and purified from serum using ExoQuick exosome precipitation solution (System Biosciences, EXOQ20A) according to the manufacturer’s instructions.

The protein content was measured using a BCA protein assay (Thermo Scientific). Atrial myocytes-derived exosomes were analyzed for the presence of the exosomal marker proteins ALIX, CD63 and CD81 using Western blot, and the relative expression levels of miR-210-3p and exosomal miR-210-3p were determined using qRT-PCR. For exosome uptake experiments, exosomes were observed and imaged using a Philips CM12 electron microscope (FEI Company) operated at 60–120 kV and equipped with a digital camera. Atrial myocyte-derived exosomes were labeled with the PKH67 Green Fluorescent Cell Linker Kit (Sigma, Aldrich) according to the manufacturer’s protocol.

### Transfection

Atrial myocytes were transfected with mimic, inhibitor and (negative control) NC of miR-210-3p using Lipofectamine 2000, and the culture supernatant was collected for exosome isolation. The miR-210-3p mimic, miR-210-3p inhibitor, siRNA-GPD1L and siRNA-NC were synthesized by GenePharma (Shanghai, China). The transfected atrial myocytes were subjected to tachypacing for 24 h, and an atrial myocyte pacing culture system (C-PACE100™, Ionoptix Corp, Milton, MA) was used to culture and pace the cells with a pacing frequency of 5 Hz.

### Cell counting kit-8 assay

The CCK-8 assay was performed by inoculating atrial fibroblasts into 96-well plates. Then, the exosomes or reagent-containing medium was added to the cells for further culture (field preparation when in use). After 24 h culture in the incubator, 10 μl CCK-8 reagent (Sigma, USA) were added to the fresh medium of each well. the absorbance at 450 nm was measured by an enzyme-labeled instrument. The absorbance was measured once every 0.5 h and 4 times for 3 days.

### Immunofluorescence staining

Atrial fibroblasts were fixed with 4% paraformaldehyde for 20 min at room temperature and then permeabilized with 0.5% Triton X-100 for 20 min at room temperature. The primary antibodies used in this experiment were incubated at 4 ℃ overnight, as follows: alpha-smooth muscle actin (α-SMA) antibody (1:100, Abcam, US) and then incubated with the following secondary antibodies (Beyotime, China, 1:200) for 90 min at room temperature. Nuclei were stained with DAPI (Beyotime, China). Cells were observed using a laser scanning confocal microscope (100x, ZEISS 510S, Germany).

### Quantitative reverse transcription-PCR (qRT-PCR)

Total RNA was extracted with RNA extraction kit (Axygen, USA) according to the manufacturer’s instructions. The rnomiR-210-3p and Collagen I, α-SMA, and TGFβ1 mRNA levels were determined using a standard SYBR Green PCR kit (Roche, Switzerland) and an Applied Biosystems 7500 Real-Time PCR System (Applied Biosystem, USA). GAPDH (for mRNAs) and U6 (for miRNAs) were used as internal controls. GPD1L-specific primers were obtained from Comate Bioscience. The miR-210-3p-, miR-449-, miR-200a-, miR-320-, miR-22-, and U6-specific primers were prepared using Bulge-Loop miRNA qRT‒PCR primers (RiboBio). Data were analyzed using the comparative 2^(−ΔΔCT)^ method to quantify relative gene expression. The mRNA primers are listed in Additional file 1: Table S1.

### Western blot analysis

Total protein was extracted from cultured atrial fibroblasts or myocardial tissues, and the concentrations of the proteins were determined using a BCA Protein Assay Kit. Equal concentrations of proteins were resolved on 10% SDS-PAGE gels and subsequently transferred to PVDF membranes. After blocking with 5% skim milk for 1.5 h, the membranes were incubated with primary antibodies against ALIX (1:500, Abcam, ab232611), CD63 (1:500, Abclonal, A5271), CD81 (1:500, Abcam, ab109201), α-SMA (1:1000, Abcam, ab124964), Collagen I (1:1000, Abcam, ab260043), Collagen III (1:1000, Proteintech, 22,734–1-AP), GPD1L (1:1000, Proteintech, 17,263–1-AP), PI3K (1:1000, CST, 4249), AKT (1:1000, CST, 9271S), PAKT (1:1000, CST, 473S) and GAPDH (1:1000, Cell Signaling Technology, 97,166) at 4 °C overnight. The membranes were then incubated with a secondary antibody at room temperature for 1 h. Chemiluminescent signals were developed with an ECL kit and detected using a ChemiDoc XRS gel documentation system (Bio-Rad, Hercules, CA, USA). The results are reported as fold changes after normalizing the data to the control values.

### Dual-Luciferase assay

Target genes for miR-210-3p were predicted and overlapped using three algorithms: TargetScan, miRanda, and miRDB. For luciferase assays, HEK293 cells were cultured in 6-well plates and cotransfected with wild-type or mutant GPD1L 3′-UTR reporters (0.1 μg) and a miR-210-3p expression plasmid or empty vector. Luciferase activities were measured 48 h after transfection using the Dual-Luciferase reporter assay system (Promega) according to the manufacturer’s instructions.

### RNA-Seq analysis

The exosomal miRNA-seq analysis was performed using the BGISEQ-500 platform (BGI-Shenzhen, China). Furthermore, DEG-seq was used to analyze the differentially expressed miRNAs among all groups. A *P* value < 0.05 and log2 (fold change) > 1 were considered statistically significant. Then, atrial fibroblasts were transfected with si-NC or si-GPD1L, and WT or KO rat hearts were treated according to the manufacturer’s protocol. The mRNA-seq analysis was performed using the Illumina platform (Illumina, USA) with paired-end reads of 150 bp at RiboBio Co., Ltd. (Ribobio, China). The differentially expressed genes were identified based on an adjusted P value < 0.05 and log2 (fold change) > 1 using edge R software.

### GO and KEGG analyses

The differentially expressed mRNAs were analyzed using Gene Ontology (GO) and Kyoto Encyclopedia of Genes and Genomes (KEGG) pathway databases. The GO analysis included the molecular functions, cellular components and biological processes of genes. The biological functions of these genes were further annotated by KEGG pathways. A *P* value < 0.05 was considered statistically significant.

### Statistical analysis

All data were analyzed using GraphPad Prism 7.0 software. Continuous variables are presented as means ± standard deviation. The significance of differences between groups was evaluated using an unpaired Student’s t test, and the differences between multiple groups were analyzed using one-way ANOVA followed by Tukey’s tests. A *P* value < 0.05 was considered statistically significant. The statistical analysis of clinical characteristics involved in the human study was performed using SPSS 17.0. Categorical variables are presented as numbers and percentages. If the values displayed a normal distribution, the independent sample t test was used. Otherwise, the nonparametric Kruskal–Wallis test was used.

## Results

### Characterization of atrial myocyte-derived exosomes and the effect of exosomal miRNAs

**Fig. 1:**
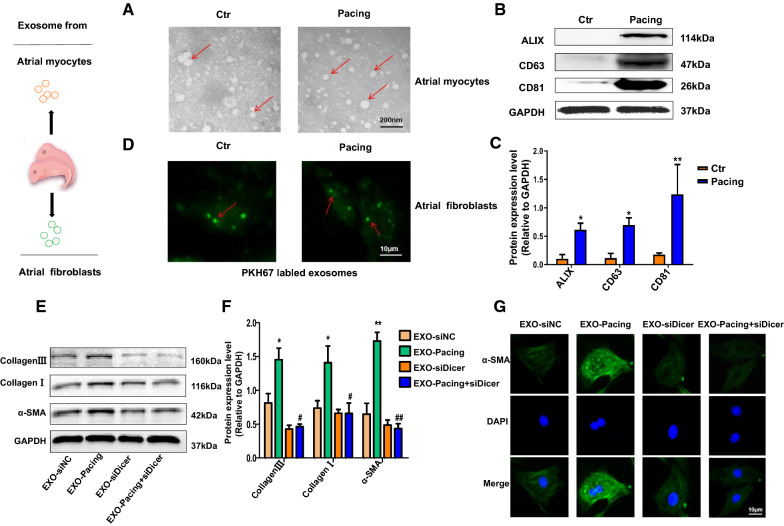
Atrial myocytes secrete exosomes and exert a profibrotic effect through their miRNA cargo. **A** Electron microscopy image of atrial myocyte-derived exosomes. The image shows small vesicles approximately 100 nm in diameter; scale bar: 200 nm. **B**, **C** Western blot characterization of atrial myocyte-derived exosomes based on the expression of the exosomes markers ALIX, CD63 and CD81 (*P < 0.05 and **P < 0.01 compared with the Ctr group). **D** Confocal imaging analysis of exosomes uptake experiment. Purified atrial myocyte-derived exosomes were labeled with PKH67 (green), and incubated with atrial fibroblasts. Scale bar: 10 μm. **E**, **F** Western blot analysis of the expression levels of collagen I, collagen IIIand α-SMA in atrial fibroblasts treated with atrial myocytes-derived exosomes. (*P < 0.05 and **P < 0.01 compared with EXO-siNC; #*P* < 0.05 and ##*P* < 0.01 compared with EXO-Pacing). **G** Immunofluorescence staining showing the relative expression levels of α-SMA (green) in atrial fibroblasts; nuclei were stained with DAPI (blue). Scale bar: 10 μm

Previous studies have shown that exosomes play critical roles in cardiac fibroblast proliferation and angiogenesis [16]. Exosomes contain and transport miRNAs associated with various intercellular communications. Exosomes were isolated from conditioned media collected from control and tachypacing atrial myocytes, and the obtained fraction was characterized using transmission electron microscopy to assess the potential role of atrial myocyte-derived exosomes in rats (Fig. 1A). Transmission electron micrographs showed that conditioned media from atrial myocytes contained predominantly exosome-sized (< 100 nm in diameter) particles. Western blot confirmed the presence of the exosome-associated protein markers ALIX, CD63 and CD81. Furthermore, the number of exosomes present in conditioned media of tachypacing atrial myocytes was significantly increased compared with the control group (Fig. 1B, C). Finally, we investigated whether exosomes were transferred between atrial myocytes and atrial fibroblasts. Exosomes were isolated from conditioned media of atrial myocytes and labeled with the fluorescent membrane marker PKH67. Exosomes (2 µg) were cocultured with atrial fibroblasts for analysis using confocal microscopy (Fig. 1D). Atrial myocyte-derived exosomes were transferred into atrial fibroblasts. We transfected atrial myocytes with si-Dicer enzyme and isolated exosomes, and 2 µg of exosomes were added to 1 × 10^5^ recipient atrial fibroblasts and incubated for 48 h to test the potential function of atrial myocyte-derived exosomal miRNAs. We also assessed the levels secreted collagen I and collagen IIIand the mesenchymal cell marker α-SMA. The levels of the collagen I, collagen IIIand α-SMA proteins were decreased in the group transfected with si-Dicer compared to the tachypacing group (Fig. 1E, F). Immunofluorescence staining showed a decrease in the expression of the fibrotic marker α-SMA in the si-Dicer treatment group. However, the opposite effects on the expression of these proteins were observed in the tachypacing group (Fig. 1G). As a result, miRNAs may play critical roles in the profibrotic process. We also verified that atrial fibroblasts treated with exosomes secreted from tachypacing atrial myocytes displayed significantly increased fibrotic protein expression levels compared with untreated atrial fibroblasts (Additional file 2: Figure S1A, B). At the same time, we also suggested that the expression levels of fibrotic protein was significantly increased in atrial fibroblasts compared with the atrial myocytes (Additional file 2: Figure S1C, D).

### Sequence analysis of miRNA expression profiles in exosomes derived from atrial myocytes and serum from patients with AF

**Fig. 2:**
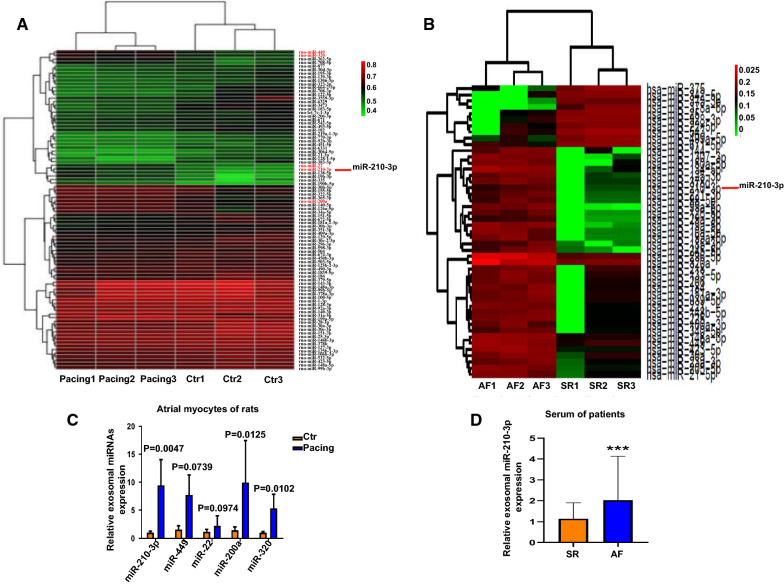
miRNA profiles of atrial myocytes and patient serum-derived exosomes. **A** Sequencing of differentially expressed exosomal miRNAs between the tachypacing and control groups of atrial myocytes. **B** Sequencing of differentially expressed exosomal miRNAs between AF and SR groups of patients. **C** qRT‒PCR of selected differentially expressed miRNAs in atrial myocyte-derived exosomes. **D** miR-210-3p was detected in exosomes of patient serum (***P < 0.001 compared with the SR group)

We profiled miRNA sequences to identify differentially expressed miRNAs in exosomes that might account for their functions related to the progression of AF. Exosomes were purified from control primary atrial myocytes and tachypacing primary atrial myocytes (5 Hz, 24 h). We identified 91 differentially expressed miRNAs and 41 upregulated miRNAs (Fig. 2A). Furthermore, serum exosomes were purified from 3 patients with SR and 3 patients with AF, and 78 miRNAs were differentially expressed (Fig. 2B). Five miRNAs were upregulated in exosomes of atrial myocytes and serum from patients with AF: miR-22, miR-200a, miR-449 miR-320 and miR-210-3p. These 5 differentially expressed miRNAs were selected and analyzed using qRT-PCR to confirm the sequencing results (Fig. 2C). Subsequently, we showed that EXO-Pacing + miR-200a inhibitor did not significantly inhibit atrial fibroblast activation and collagen deposition compared with the EXO-Pacing group (Additional file 2: Figure S2A, B). Furthermore, the GO enrichment analysis of the differentially expressed exosomal miR-210-3p derived from atrial myocytes revealed the enrichment of proteins related to fibroblast growth factor-activated receptor activity (Additional file 2: Figure S3A, B). The miR-210-3p expression level was significantly increased in exosomes from tachypacing atrial myocytes compared with the control group. Moreover, we collected serum from patients, and the clinical characteristics are presented in Additional file 1: Tables S2-S4. Similar results were obtained for exosomes from patient serum (Fig. 2D). Compared with the SR group, the atrial diameter of patients with AF was significantly increased (Additional file 1: Table S3). We detected miR-210-3p expression levels in atrial myocytes, atrial fibroblasts and exosomes to determine whether miR-210-3p was derived from atrial myocytes and transferred into atrial fibroblasts by exosomes. The results showed significant increases in miR-210-3p expression in both atrial myocytes and atrial myocyte-derived exosomes (Additional file 2: Figure S4A, B). However, the expression levels of miR-210-3p in atrial fibroblasts and atrial fibroblast-derived exosomes were not significantly different from those in the control group (Additional file 2: Figure S4C, D). In addition, atrial myocyte-derived exosomes were cocultured with conditioned medium from atrial fibroblasts. This finding indicates that miR-210-3p is transported between atrial myocytes and atrial fibroblasts by exosomes (Additional file 2: Figure S4E).

### Atrial myocyte-derived exosomes promote atrial fibroblast proliferation and collagen synthesis, an effect that is attenuated by a miR-210-3p inhibitor

Atrial fibrosis controls the development of AF by regulating structural remodeling [17]. The mechanisms leading to atrial fibrosis are complex and are specifically involved in the activation of atrial fibroblasts into myofibroblasts [18]. We first verified the transfection efficiency of the miR-210-3p mimic and inhibitor to determine whether exosomal miR-210-3p has important functions, and the results are shown in Additional file 2: Figure S4F. We transfected atrial myocytes with the miR-210-3p inhibitor for 24 h and then subjected atrial myocytes to tachypacing for 24 h. Next, 2 µg of exosomes were added to 1 × 10^5^ recipient atrial fibroblasts and incubated for 48 h before a cell proliferation assay was conducted. We also evaluated the expression levels of the fibrotic marker TGFβ1, secreted Collagen I, and mesenchymal cell marker α-SMA. We found that atrial fibroblasts treated with post-tachypacing exosomes exhibited significantly increased mRNA (Additional file 2: Figure S4G-I) and protein (Additional file 2: Figure S5A-C) levels of α-SMA, Collagen I, and TGFβ1, while atrial fibroblasts treated with the exosome inhibitor exhibited significantly decreased mRNA (Additional file 2: Figure S4G-I) and protein levels (Additional file 2: Figure S5A-C). The proliferation rate of atrial fibroblasts was analyzed by performing a CCK-8 assay, and atrial fibroblasts treated with miR-210-3p inhibitor displayed decreased viability (Additional file 2: Figure S5D). Conversely, atrial fibroblasts treated with postpacing exosomes exhibited increased cell viability. Similar results were obtained from the immunofluorescence analysis (Additional file 2: Figure S5E). Based on these results, exosomal miR-210-3p plays a critical role in the profibrotic process.

### AgomiR-210-3p exacerbates atrial fibrosis and AF development in rats

**Fig. 3:**
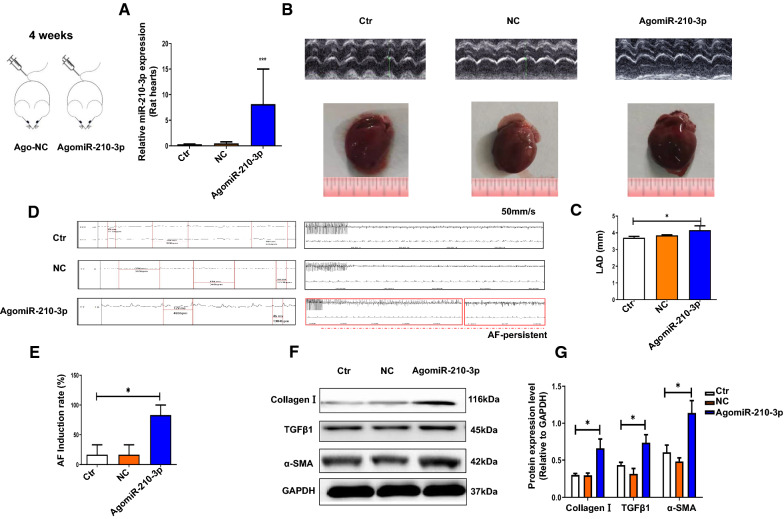
Effects of AgomiR-210-3p on atrial function and structure in rats. **A** qRT-PCR analysis of the expression of miR-210-3p in the rats heart (n = 7; ***P < 0.001 compared with the Ctr and NC groups). **B** Representative echocardiographic images captured 4 weeks after the AgomiR-210-3p infusion treatment. **C** The LAD were calculated at 4 weeks in rats (n = 7; *P < 0.05). **D** Representative electrocardiogram (ECG) traces after atrial burst pacing (left) at 3000 beats/min. **E** Probability of AF induction by atrial burst pacing of the induced AF episodes (n = 7; *P < 0.05). **F**, **G** TGFβ1, α-SMA and Collagen I protein levels were determined in atrial tissues (n = 7; *P < 0.05)

We detected miR-210-3p levels in the left atrium of rat hearts to investigate whether atrial myocyte-derived exosomal miR-210-3p is crucially involved in atrial fibrosis development, and miR-210-3p expression was significantly increased in the left atrium of rats treated with AgomiR-210-3p compared with that in control rats (Fig. 3A). Furthermore, we evaluated cardiac structure and function using echocardiography (Fig. 3B, C). Compared with the control and NC groups, LAD was increased in scrambled AgomiR-210-3p-injected rats. The ECG recordings of rats obtained during atrial electrophysiology studies are shown in Fig. 3D. Consistently, the average incidence of AF was increased in rats injected with AgomiR-210-3p compared to the control and NC groups (Fig. 3E). We also assessed the degree of atrial fibrosis by evaluating the expression levels of the fibrotic marker TGFβ1, the secreted Collagen I, and the mesenchymal cell marker α-SMA. Western blot (Fig. 3F, G) showed increased levels of the TGFβ1, α-SMA and Collagen I proteins in the AgomiR-210-3p group. These findings revealed that AgomiR-210-3p promotes atrial fibrosis leading to AF in rats.

### Knockout of miR-210-3p prevents Ang II-induced AF occurrence and persistence by attenuating atrial fibrosis in rats

**Fig. 4:**
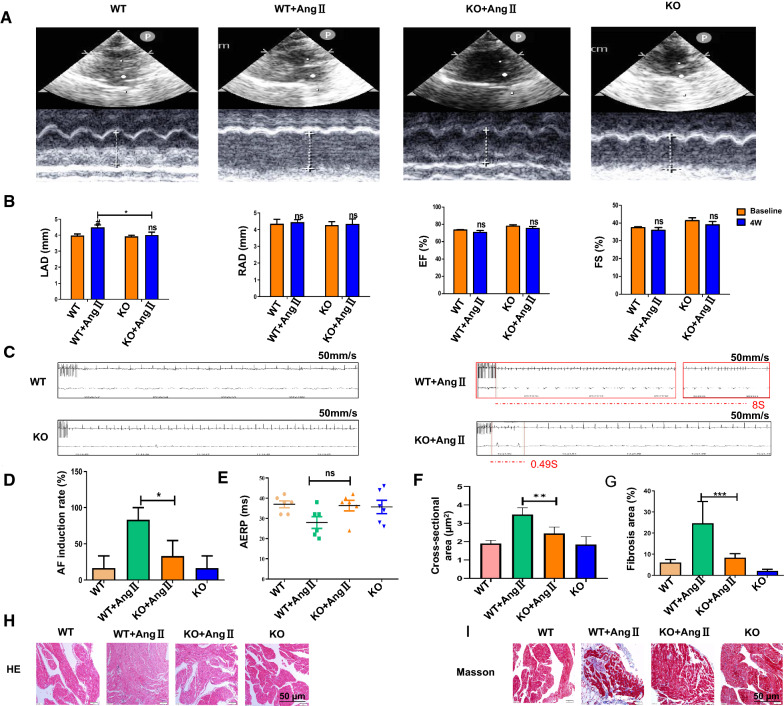
Effect of miR-210-3p KO on cardiac features and function in Ang II-infused rats. **A** Representative echocardiographic images captured 4 weeks after the Ang II infusion in rats that received KO-miR-210-3p treatment. **B** The LAD was calculated at 4 weeks in rats (n = 7; *P < 0.05 for the comparison of the WT + Ang II and KO + Ang II groups). The EF%, FS% and RAD calculated at 4 weeks in rats (n = 7; ns for the comparison between the WT + Ang II and KO + Ang II groups). **C** Representative ECG traces after atrial burst pacing (left) at 3000 beats/min. **D** Probability of AF induction by atrial burst pacing of the induced AF episodes (n = 7; *P < 0.05 for the comparison of the WT + Ang II and KO + Ang II groups). **E** AERP measurement in rats (n = 7; ns for the comparison of the WT + Ang II and KO + Ang II groups). **F** The atrial myocyte cross-sectional area was calculated with Image J software. Scale bar: 50 μm (n = 7; **P < 0.01). **G** Atrial fibrosis (the ratio of the fibrotic area to total atrium area) was measured using Image J software. Scale bar: 50 μm (n = 7; ***P < 0.001). **H** Representative images of HE staining of atrial samples; the results are graphed for representative bright field images. **I** Representative images of Masson’s trichrome staining of atrial samples

We identified whether administration of KO-miR-210-3p decreased Ang II infusion-induced atrial fibrosis in vivo to further confirm the role of miR-210-3p in the development of cardiac fibrosis. We evaluated cardiac structure and function using echocardiography (Fig. 4A). As shown in Fig. 4B, the LAD was substantially increased in rats from the WT + Ang II group compared with the KO-miR-210-3p + Ang II group. We also assessed the levels of EF% and FS%, but the differences were not significant. Thus, miR-210-3p KO ameliorates the atrial dysfunction induced by Ang II infusion. The ECG recordings of the rats during electrophysiology studies are shown in Fig. 4C. Consistently, the incidence of AF was increased in rats treated with Ang II compared to the KO-miR-210-3p + Ang II group (Fig. 4D). In addition, the duration of AERP is shown in Fig. 4E; however, the difference was not significant. Furthermore, the AF induction rates were significantly increased in the WT + Ang II group compared with the KO + Ang II group. Collagen deposition was assessed using Masson's trichrome staining and HE staining, and the fibrotic areas of WT + Ang II rats were significantly increased compared with those of KO + Ang II rats (Fig. 4F−I). qRT-PCR results showed that miR-210-3p expression decreased in the heart, liver, spleen, and kidney of KO rats (Additional file 2: Figure S6A). The Western blot (Additional file 2: Figure S6B, D), qRT-PCR (Additional file 2: Figure S6C) and immunohistochemistry (Additional file 2: Figure S6E, F) results showed that miR-210-3p KO downregulated fibrosis-related protein and mRNA expression levels in rats. miR-210-3p KO in hearts treated with Ang IIto induce atrial fibrosis reversed the Ang II-induced profibrotic effect, suggesting a potential therapeutic use.

### GPD1L is a target of miR-210-3p in atrial fibroblasts

**Fig. 5:**
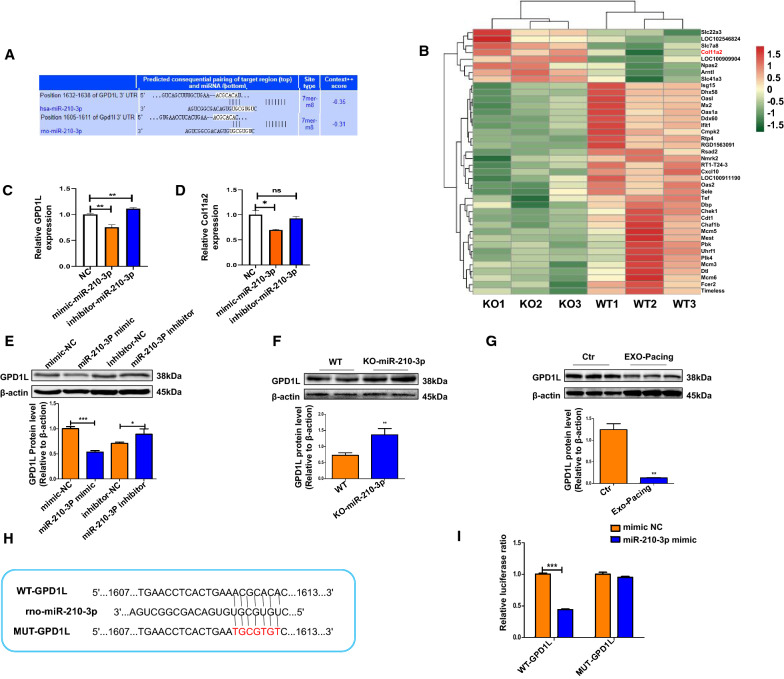
GPD1L is a target of miR-210-3p and may mediate its effects on fibrosis. **A** The site predicted GPD1L as a target gene. **B** RNA-Seq analysis of the differentially expressed mRNAs in the WT and miR-210-3p KO groups of rat hearts. Levels of the GPD1L (**C**) and Col11a2 (**D**) mRNAs were analyzed in atrial myocytes by qRT-PCR (*P < 0.05 and **P < 0.01). **E** The GPD1L protein expression level in the mimic and inhibitor groups of atrial myocytes (*P < 0.05 compared with the inhibitor NC; ***P < 0.001 compared with the mimic NC). **F** The GPD1L protein expression level in WT and KO groups of rats (n = 7; *P < 0.05). **G** The GPD1L protein expression level in atrial fibroblasts treated with atrial myocyte-derived exosomes in Ctr and EXO-Pacing groups (***P < 0.001). **H** Wild-type sequence (WT-GPD1L) and mutated sequence (MUT-GPD1L) for the miR-210-3p binding site. **I** Dual-luciferase expression is shown for GPD1L (***P < 0.001 compared with mimic NC)

Exosomal miRNAs regulate the atrial function and structure by directly targeting genes [10]. We utilized TargetScan, miRanda and miRDB to identify the potential binding sites for miR-210-3p and investigate the molecular mechanism underlying the effects of miR-210-3p on AF; among them, glycerol-3-phosphate dehydrogenase 1-like (GPD1L) was strongly silenced and was a very interesting candidate (Fig. 5A). Additionally, small RNAs were extracted and analyzed by RNA-Seq, the expression of 8 genes was upregulated in atrial tissue of KO rats, Col11a2 was associated with cardiac fibrosis (Fig. 5B). qRT-PCR showed that the binding of GPD1L with miR-210-3p have a more significant difference compared with Col11a2 in atrial myocytes, as shown in Fig. 5C and D. Furthermore, GPD1L protein expression levels were downregulated in the mimic-miR-210-3p group, but were upregulated in the inhibitor-miR-210-3p group compared to the NC group in atrial myocytes (Fig. 5E). The GPD1L protein expression levels were also detected in rats, and the results showed that GPD1L protein expression was upregulated in the KO-miR-210-3p group compared to the WT group (Fig. 5F). In addition, we treated tachypacing atrial myocytes-derived exosomes with conditioned medium from atrial fibroblasts, and GPD1L expression was significantly downregulated in the Exo-Pacing group compared with the control group (Fig. 5G). Based on these results, miR-210-3p affects GPD1L expression by blocking translation. The wild-type sequence (WT-GPD1L) and mutated sequence (MUT-GPD1L) of the putative miR-210-3p binding site in the GPD1L 3’UTR were cloned into a luciferase vector to confirm whether GPD1L is a target gene of miR-210-3p (Fig. 5H). A dual-luciferase reporter assay showed that luciferase activity was significantly upregulated after cotransfection with the miR-210-3p mimic and the reporter vector containing the wild-type GPD1L promoter, while no significant change was observed with the mutant GPD1L promoter (Fig. 5I).

### miR-210-3p promotes the proliferation of and collagen synthesis by atrial fibroblasts by targeting GPD1L

**Fig. 6:**
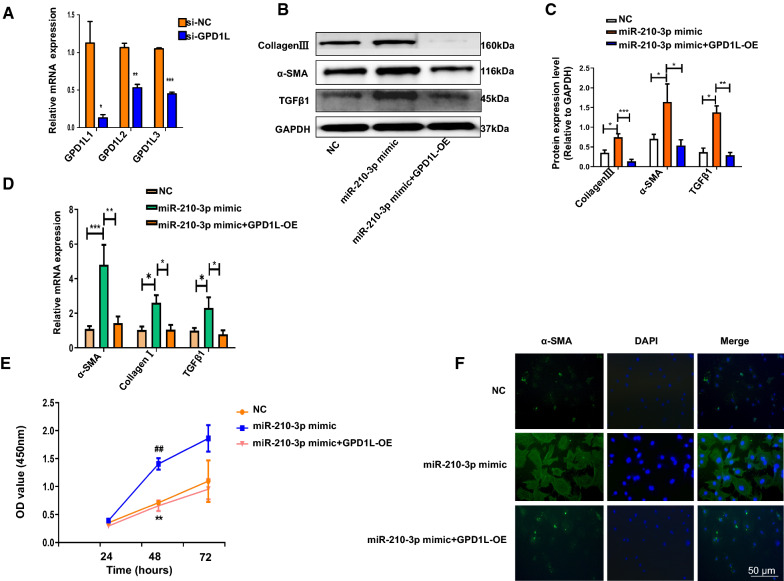
miR-210-3p promotes fibroblast proliferation and collagen synthesis by targeting GPD1L. **A** GPD1L was silenced by siRNAs in atrial fibroblasts. The protein **B**, **C** expression levels of Collagen III, TGFβ1 and α-SMA and mRNA (**D**) expression levels of α-SMA, Collagen I and TGFβ1 in atrial fibroblasts (*P < 0.05, **P < 0.01, and ***P < 0.001). **E** CCK8 assay to detect the effect of miR-210-3p and GPD1L on the proliferation of atrial fibroblasts (##*P* < 0.05 compared with NC, **P < 0.05 compared with miR-210-3p mimic + GPD1L). **F** Immunofluorescence staining to detect the relative expression levels of α-SMA (green) and DAPI (blue) in atrial fibroblasts. Scale bar: 50 μm

GPD1L, also known as glyceraldehyde-3-phosphate dehydrogenase, plays an important role in metabolism. Previous studies have shown that GPD1L mutations can cause arrhythmias such as Brugada syndrome [19]. GPD1L can significantly inhibits cell proliferation and migration and promotes cell apoptosis [20]. We explored whether miR-210-3p promoted atrial fibroblast proliferation and activation by directly targeting GPD1L. First, we analyzed the expression levels of fibrotic proteins (Additional file 2: Figure S8A, B) and mRNAs (Additional file 2: Figure S8C) in the si-NC and si-GPD1L groups. Then, we silenced GPD1L in atrial fibroblasts using siRNAs (Fig. 6A). GPD1L-OE (plasmid overexpressing GPD1L) and the miR-210-3p mimic were transfected in atrial fibroblasts for 24 h. Moreover, the protein (Fig. 6B, C) expression of Collagen III, TGFβ1 and α-SMA was significantly upregulated in the miR-210-3p mimic group compared with the miR-210-3p mimic + GPD1L-OE group. The expression of the α-SMA, collagen I and TGFβ1 mRNAs was upregulated in the miR-210-3p mimic group compared with the miR-210-3p mimic + GPD1L-OE group (Fig. 6D). CCK-8 assays showed that the miR-210-3p mimic substantially increased cell viability compared to the miR-210-3p mimic + GPD1L-OE group (Fig. 6E). Changes in α-SMA expression were further verified by immunofluorescence staining (Fig. 6F). In general, our results indicate that miR-210-3p regulates the atrial fibroblast proliferation, activation and collagen synthesis by targeting GPD1L.

### GPD1L regulates atrial fibroblast proliferation and activation via the PI3K/AKT pathway

**Fig. 7:**
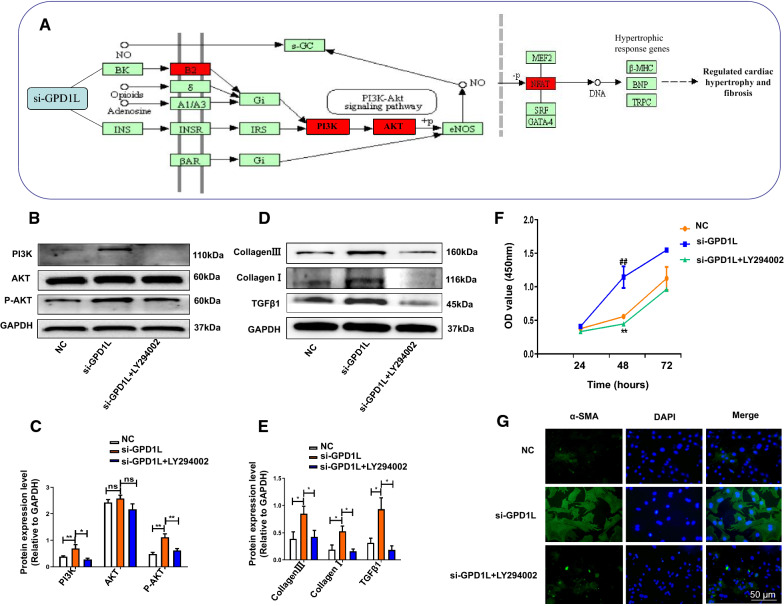
The miR-210-3p-mediated GPD1L/PI3K/AKT signaling pathway contributes to fibrosis and the changes are reversed by LY294002. **A** KEGG pathway analysis of signaling molecules downstream of GPD1L. **B**, **C** Western blot analysis of the levels of the PI3K, P-AKT and AKT proteins in atrial fibroblasts (*P < 0.05, **P < 0.01, and ***P < 0.001). **D**, **E** Western blot analysis of the levels of the TGFβ1, Collagen I and Collagen III proteins in atrial fibroblasts (*P < 0.05). **F** CCK-8 assay of atrial fibroblast proliferation (##*P* < 0.01 compared with NC and **P < 0.01 compared with si-GPD1L + LY294002). **G** Immunofluorescence staining showed the relative expression levels of α-SMA (green) and DAPI (blue) in atrial fibroblasts. Scale bar: 50 μm

We performed RNA-Seq (Additional file 2: Figure S7A, B) and KEGG pathway (Fig. 7A) analyses to determine whether the PI3K/AKT signaling pathway was associated with GPD1L-mediated fibrosis and investigate the molecular mechanism underlying the effects of miR-210-3p on atrial fibroblast proliferation and activation. GPD1L-OE and mimic-miR-210-3p were transfected into atrial fibroblasts for 24 h. Western blot analysis suggested that GPD1L-OE treatment significantly decreased the levels of PI3K and AKT phosphorylation (Figure S8D-F). Atrial fibroblasts were treated with the PI3K inhibitor LY294002 (20 μmol/L) 2 h prior to transfection. Western blot assays showed that treatment with LY294002 decreased the levels of the PI3K, AKT and P-AKT proteins compared with the si-GPD1L group (Fig. 7B, C), and the Collagen III, Collagen I and TGFβ1 protein levels (Fig. 7D, E) were decreased in the si-GPD1L + LY294002 group compared with the si-GPD1L group. Moreover, the mRNA (Additional file 2: Figure S8G-I) expression levels of α-SMA, collagen I and TGFβ1 were decreased in the si-GPD1L + LY294002 group compared with the si-GPD1L group. In addition, CCK-8 assays showed that cell viability was substantially decreased in the si-GPD1L + LY294002 group compared with the si-GPD1L group (Fig. 7F). Immunofluorescence staining showed a decrease in the expression of the fibrosis marker α-SMA in the si-GPD1L + LY294002 group (Fig. 7G). These findings confirm that exosomal miR-210-3p promotes atrial fibrosis-induced AF by targeting the GPD1L/PI3K/AKT pathway.

## Discussion

In this study, we identified a novel exosomal miRNA-mediated mechanism for communication between atrial myocytes and atrial fibroblasts. Considerable advances have been achieved toward an understanding of the role of exosomal miR-210-3p as a key molecule targeting GPD1L to promote fibroblast proliferation and excess collagen deposition via the PI3K/AKT signaling pathway and regulate adverse atrial remodeling in individuals with AF (Graphical Abstract).

Atrial fibrosis is the most crucial substrate to induce structural remodeling, which is considered the main cause of AF perpetuation [21]. Fibroblasts are the largest cell population in atrial tissues, and fibroblasts are activated into myofibroblasts [22]. These myofibroblasts secrete large amounts of extracellular matrix and show increased levels of migration [23]. Excessive deposition of extracellular matrix regulates fibroblast proliferation, migration and differentiation, which profoundly impair electrical conduction and exacerbate cardiac fibrosis related to heart failure and arrhythmias [24]. Paracrine mechanisms have been shown to regulate the crosstalk between fibroblasts and myocytes and may be associated with collagen synthesis during myocardial hypertrophy [25]. Exosomes are crucial factors involved in the process of fibrosis that regulate fibroblast proliferation and differentiation [26]. Our findings further indicate that exosomes are secreted from atrial myocytes and are transported to neighboring cells. The use of targeted treatments for AF is an intriguing approach, yet it has been challenging in recent years [27, 28]. Therefore, studies exploring the regulatory mechanisms underlying atrial fibrosis are very important for the control of AF development. Here, we found that atrial myocytes produced and secreted exosomes enriched with miRNAs. Exosomal miRNAs mediated the pathological communication between atrial myocytes and atrial fibroblasts related to AF development. Our study will provide new insights into exosome-miRNA-based therapy for AF.

Exosomes are small single-membrane vesicles with a diameter of 30–200 nm that are enriched in selected proteins, lipids, nucleic acids, and glycoconjugates and play important roles in multiple aspects of human health and disease [29, 30]. Exosomes play a critical role in cardiac repair after myocardial infarction and might bridge a major gap after myocardial injury [31]. Based on accumulating evidence, exosomes affect cardiomyocyte apoptosis and cell viability and regulate the electric and structural functions in individuals with AF [32, 33]. However, the molecular mechanism of atrial myocyte-derived exosomal miRNAs in AF has rarely been studied. Here, we examined the role of exosome-derived miRNAs in regulating atrial fibroblast proliferation, activation and collagen synthesis during AF in vitro and in vivo. These findings suggest that exosomal miRNAs may represent novel biomarkers to predict the progression of AF and aid in the identification of novel therapeutic targets to reduce AF-related mortality.

miRNAs are small noncoding RNAs that regulate gene expression by repressing translation and accelerating target mRNA degradation. These molecules are integral to almost all known biological processes, including cell growth, proliferation and differentiation, as well as organismal metabolism and development [34]. miRNAs have recently emerged as paracrine signaling mediators associated with dysfunctional gene expression profiles related to many cardiovascular disease conditions [8, 35]. Several miRNAs play critical roles in hypertrophic and fibrotic myocardial tissues, suggesting an association between specific miRNA levels and the development of pathological cardiac remodeling [36, 37]. Our in vivo and in vitro experiments showed that miR-210-3p expression is markedly increased in atrial myocyte-derived exosomes. Our studies confirmed that reducing exosomal miR-210-3p levels confer protection against pathological atrial remodeling in the context of AF by preventing atrial fibrosis and atrial fibroblast proliferation. Furthermore, using innovative technologies, as well as a gene KO rat model, we show that atrial myocytes are the major cell type that express miR-210-3p, while miR-210-3p KO effectively prevents atrial fibrosis and reduces the incidence of AF. Together, these data confirm that exosomal miR-210-3p is associated with atrial remodeling and plays a functional role in AF pathogenesis.

GPD1L has been shown to participate in cell proliferation, migration and apoptosis by regulating oxidative stress in cancer [20]. However, the function of GPD1L in AF has rarely been studied. In the present study, GPD1L silencing promoted atrial fibroblast activation and proliferation through miR-210-3p. A GPD1L mutation causes Brugada syndrome and other inherited arrhythmia syndromes by affecting Na + channel trafficking to the plasma membrane [38]. Changes in the expression of GPD1L, which are possibly mediated by the inhibition of miR-210 as a potential signaling molecule, regulate the proliferation and energy metabolism of cells in tumors [39]. According to the results from our experiments, GPD1L is a novel target for miR-210-3p in atrial fibroblasts that regulates fibroblast proliferation and activation. In addition, studies have also shown that CTGF activates the PI3K and AKT pathways, contributing to the inhibition of GPD1L expression and promoting angiogenesis in human synovial fibroblasts [40]. Zhang et al. found that the principal signaling pathways of PI3K/AKT are mainly activated in various pathological states, such as in fibrosis, apoptosis, and regeneration after myocardial infarction [41]. Upregulated PI3K and phosphorylation of AKT may be involved in the increased proliferation and migration of cardiac fibroblasts, which are reversed by a PI3K inhibitor [42]. Consistent with the results from previous studies, the fibrotic changes associated with increased miR-210-3p levels are proposed to include the regulation of GPD1L-mediated inhibition of PI3K/AKT-dependent signaling pathways. This pathway represents a previously uncharacterized interaction between miR-210-3p and GPD1L/PI3K/AKT; interestingly, these fibrotic effects were reversed by the PI3K inhibitor LY294002.

Several limitations of this study should be addressed. First, the expression of miRNAs in atrial myocyte-derived exosomes was detected in rat hearts and patient serum, but the expression of these miRNAs in atrial myocyte-derived exosomes from patient hearts may still require future experiments. Second, the present study only examined one miRNA (miR-210-3p) based on microarray data, and future studies should further explore the functions of other potential miRNAs based on microarray data. Third, the animal AF model was the atrial fibrosis model, and future studies may investigate the role of atrial myocyte-derived exosomes in other animal models.

## Conclusions

In summary, atrial myocytes treated with a miR-210-3p inhibitor exerted a protective effect on tachypacing-induced atrial fibroblast proliferation and activation. Our in vivo results indicate that miR-210-3p agonists have adverse properties that increase the AF incidence. However, miR-210-3p KO in rats with Ang II-induced atrial fibrosis prevented the development of AF. Our findings reveal that atrial myocyte-derived miR-210-3p targets the GPD1L/PI3K/AKT pathway and functions as a crucial paracrine signaling mediator during atrial fibroblast cell proliferation; we also illustrate a novel role for miR-210-3p as a potential marker for the clinical diagnosis and identification of novel therapeutic targets in AF.

## What is already known

- Exosome-mediated cellular communication may lead to cardiovascular diseases by transferring miRNAs.
- Atrial fibrosis plays a potentially important role in AF.

## What this study adds

- Exosomes mediate pathological communication between atrial myocytes and atrial fibroblasts, promoting the fibrotic response by transporting miRNAs during AF.
- Atrial myocyte-derived exosomal miR-210-3p targeted GPD1L to promote atrial fibrosis via the PI3K/AKT signaling pathway.

## What is the clinical significance

- Exosomal miR-210-3p may be a novel biomarker to predict the progression of AF.
- Exosome delivery of miRNAs is considered a novel targeted candidate drug to treat AF.

## Supplementary Information

**Additional file 1: Table S1.** The sequences of primers designed for qRT-PCR. **Table S2.** General clinical characteristics of patients. **Table S3.** Echocardiography and test results of patients. **Table S4.** Medication and surgery treatment of patients

**Additional file 2: Figure S1.** The level of fibrotic protein in atrial fibroblasts. **Figure S2.** The level of fibrotic protein in atrial fibroblasts. **Figure S3.** Bioinformatics analysis on the relationship between miR-210-3p and fibroblast activity. **Figure S4.** qRT-PCR analysis of the differentially expressed miRNAs. **Figure S5.** Exosomal miR-210-3p derived from atrial myocytes promotes atrial fibroblast proliferation and activation. **Figure S6.** Effects of miR-210-3p KO on atrial fibrosis in rats. **Figure S7.** RNA sequencing analysis. **Figure S8.** miR-210-3p promotes proliferation and collagen synthesis by targeting GPD1L/PI3K/AKT

## Data Availability

The data supporting the findings of this study are available within the article and its supplementary materials. RNA sequencing data are deposited in GEO (accession number: SE21089;https://www.ncbi.nlm.nih.gov/geo/query/acc.cgi?acc=GSE210894). All other supporting data are available from the corresponding authors on reasonable request.

## Abbreviations

AF: Atrial fibrillation
Ang II: Angiotensin II
α-SMA: Alpha-smooth muscle actin
EVs: Extracellular vesicles
EXO: Exosome
FS: Fractional shortening
GPD1L: Glycerol-3-phosphate dehydrogenase 1-like
KO: Knock out
LAD: Left atrial diameter
LVEF: Left ventricular ejection fraction
miRNA: MicroRNA
RAD: Right atrial diameter
3’ UTR: 3’ Untranslated region
TGF-β: Transforming growth factor-β
WT: Wild type
