# Supplementary material for: Atrial myocyte-derived exosomal microRNA contributes to atrial fibrosis in atrial fibrillation
Source: J Transl Med. 2022 Sep 5;20:407. doi: 10.1186/s12967-022-03617-y (PMC9446866; doi:10.1186/s12967-022-03617-y)
Supplement: Supplementary file 1 — Additional file 1: Table S1. The sequences of primers designed for qRT-PCR. Table S2. General clinical characteristics of patients. Table S3. Echocardiography and test results of patients. Table S4. Medication and surgery treatment of patients [file 12967_2022_3617_MOESM1_ESM.pdf]

Table S1. The sequences of primers designed for qRT-PCR.

Gene	Sense	Sequence
α-SMA	Forward primer	5'-GTCCCAGACATCAGGGAGTAA-3'
	Reverse primer	5'-TCGGATACTTCAGCGTCAGGA-3'
CollagenI	Forward primer	5'-GAGCGGAGAGTACTGGATCGA-3'
	Reverse primer	5'-CTGACCTGTCTCCATGTTGCA-3'
CollagenIII	Forward primer	5'-TGGCACAGCAGTCCAATGTAC-3'
	Reverse primer	5'-GACAGATCCCGAGTCGCAGA-3'
TGFβ1	Forward primer	5'-ATTCAAGTCAACTGTGGAGCAAC-3'
	Reverse primer	5'-CGAAAGCCCTGTATTCCGTCT-3'
GPD1L	Forward primer	5'-CCCCTGAAAGTGTGCATCGT-3'
	Reverse primer	5'-GGCAGCTTGTGTCCAGGAA-3'
Col11a2	Forward primer	5'-GATGAGCTGAGCCCTGAGAC-3'
	Reverse primer	5'-CCAGGTCTGAGAAGGAAGCA-3'
GAPDH	Forward primer	5'-GACATCAAGAAGGTGGTGAAGC-3'
	Reverse primer	5'-TGTCATTGAGAGCAATGCCAGC-3'

Table S2. General clinical characteristics of patients.

	Ctr patients (n=50)	AF patients (n=50)	P (value)
Age (years)	60.70±8.23	62.26±8.04	0.359
Male (n)	32	36	0.396
HBP, n (%)	28(56)	27(54)	0.842
DM, n (%)	18(36)	13(26)	0.284
CHD, n (%)	24(48)	23(46)	0.843
HF, n (%)	7(14)	10(20)	0.429
Smoke, n (%)	22(44)	26(52)	0.428

Data are presented as mean±SD, n, % or median. HBP: hypertention; DM: diabetes mellitus; CHD: coronary heart disease; HF: heart failure.

Table S3. Echocardiography and test results of patients.

	Ctr patients (n=50)	AF patients (n=50)	P (value)
TG (mmol/L)	1.69±0.79	1.91±0.76	0.379
CHOL (mmol/L)	4.66±0.83	4.19±1.07	0.079
LDL (mmol/L)	2.83±0.60	2.62±0.87	0.303
UA (umol/L)	315.0±54.58	354.4±69.63	0.246
RAD (mm)	43.29±3.03	47.95±5.50	0.020
LAD (mm)	36.12±3.44	40.27±4.42	0.003
LVEF (%)	60.82±9.36	62.82±6.59	0.440

Data are presented as mean±SD, n, % or median. TG: triglyceride; CHOL: cholesterol; LDL: low density lipoprotein; UA: uric acid; RAD: right atrial diameter; LAD: left atrial diameter; LVEF: left ventricular ejection fraction.

Table S4. Medication and surgery treatment of patients.

	Ctr patients (n=50)	AF patients (n=50)	P (value)
Antiplatelet, n (%)	23(46)	22(44)	0.842
Statins, n (%)	20(40)	23(46)	0.548
β-blocker, n (%)	19(38)	28(56)	0.072
Amiodarone, n (%)	5(10)	19(38)	0.001
NOAC, n (%)	0(0)	30(60)	＜0.001
Digoxin, n (%)	4(8)	15(30)	0.004
CCB, n (%)	20(40)	27(54)	0.164
RFA, n (%)	0(0)	19(38)	＜0.001
PCI, n (%)	17(34)	15(30)	0.671

Data are presented as mean±SD, n, % or median. NOAC: novel oral anticoagulants; CCB: calcium channel blocker; RFA: radiofrequency ablation; PCI: percutaneous cardiac intervention.
