# Supplementary material for: Atrial myocyte-derived exosomal microRNA contributes to atrial fibrosis in atrial fibrillation
Source: J Transl Med. 2022 Sep 5;20:407. doi: 10.1186/s12967-022-03617-y (PMC9446866; doi:10.1186/s12967-022-03617-y)
Supplement: Supplementary file 2 — Additional file 2: Figure S1. The level of fibrotic protein in atrial fibroblasts. Figure S2. The level of fibrotic protein in atrial fibroblasts. Figure S3. Bioinformatics analysis on the relationship between miR-210-3p and fibroblast activity. Figure S4. qRT-PCR analysis of the differentially expressed miRNAs. Figure S5. Exosomal miR-210-3p derived from atrial myocytes promotes atrial fibroblast proliferation and activation. Figure S6. Effects of miR-210-3p KO on atrial fibrosis in rats. Figure S7. RNA sequencing analysis. Figure S8. miR-210-3p promotes proliferation and collagen synthesis by targeting GPD1L/PI3K/AKT [file 12967_2022_3617_MOESM2_ESM.pdf]

Supplement figure 1

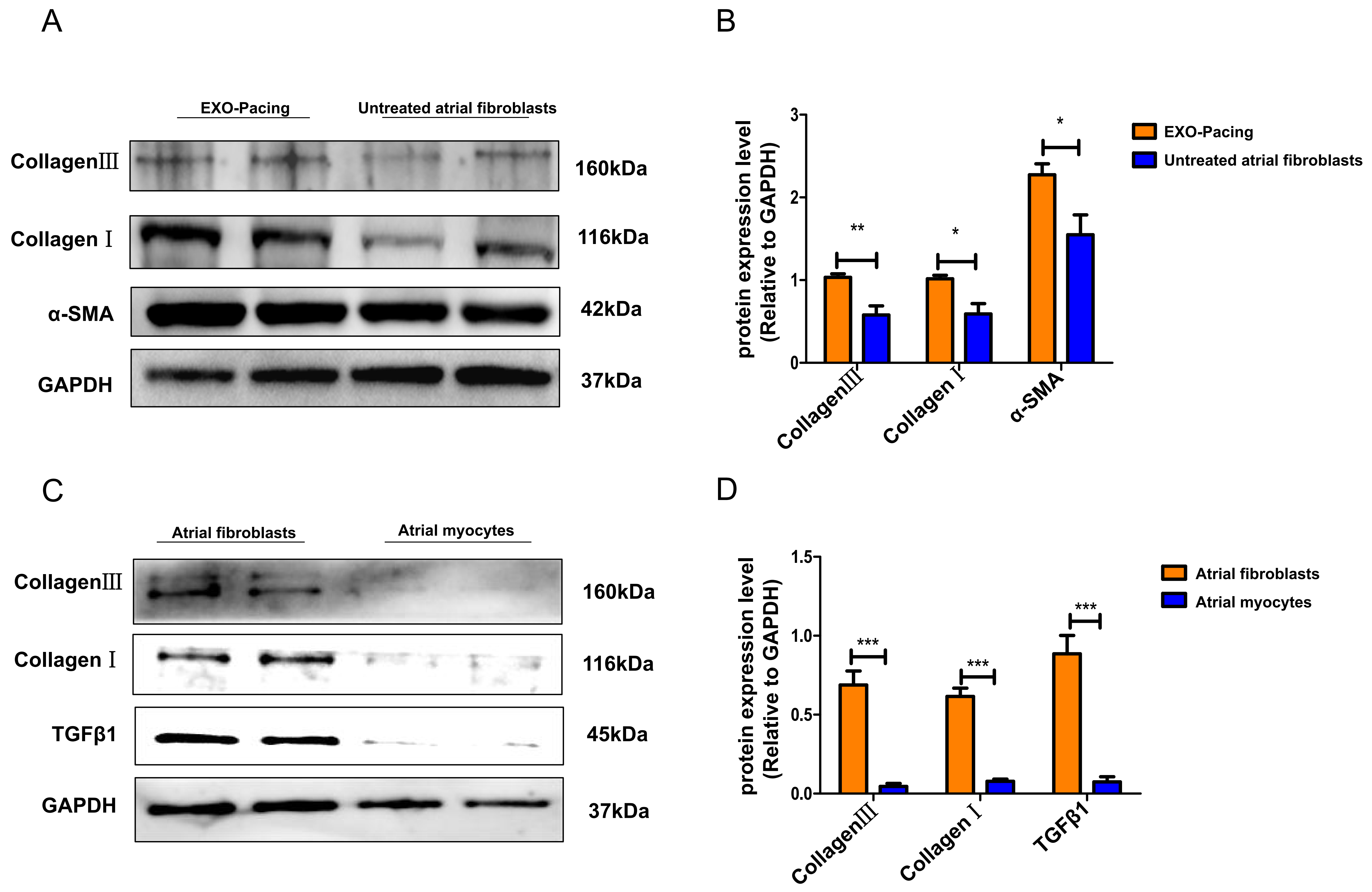

The level of fibrotic protein in atrial fibroblasts. (A, B) Western blot analysis of protein levels in EXO-Pacing and untreated atrial fibroblasts (*P<0.05 and **P<0.01). (C, D) Western blot analysis of protein levels in atrial fibroblasts and atrial myocytes. (**P*<0.05, ****P*<0.001). The levels of fibrotic proteins in atrial fibroblasts.

Supplement figure 2

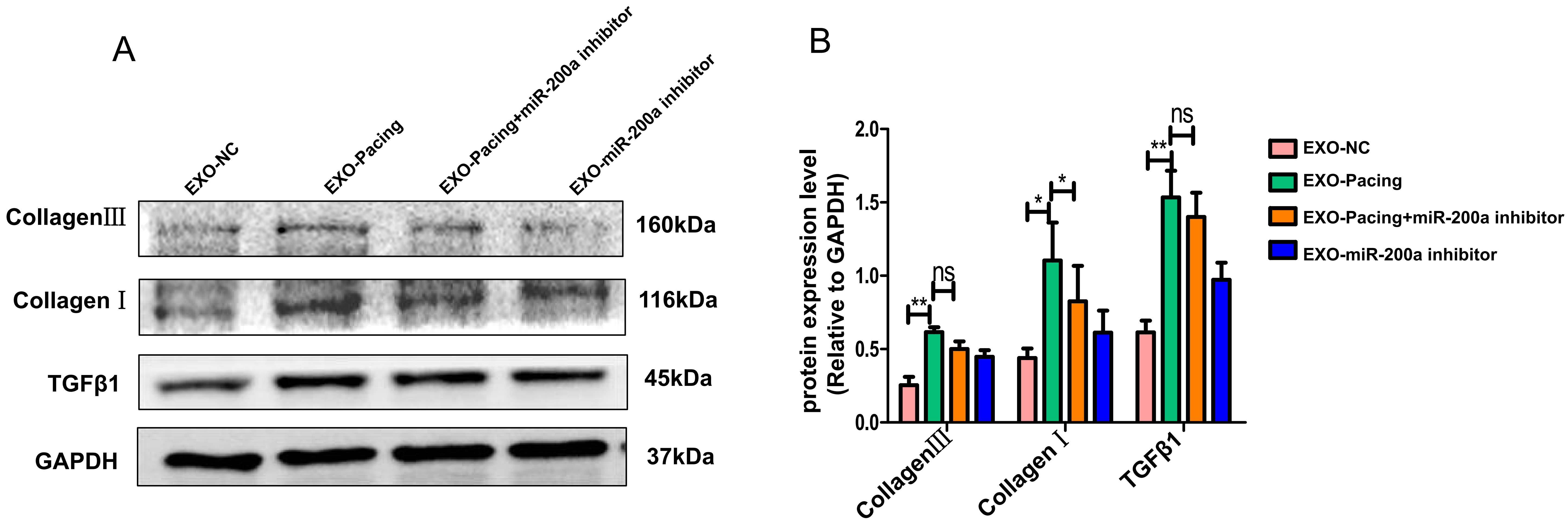

The level of fibrotic protein in atrial fibroblasts. (A, B) Western blot analysis of protein levels in atrial fibroblasts treated with exosomes derived from atrial myocytes (*P<0.05, **P<0.01).

Supplement figure 3

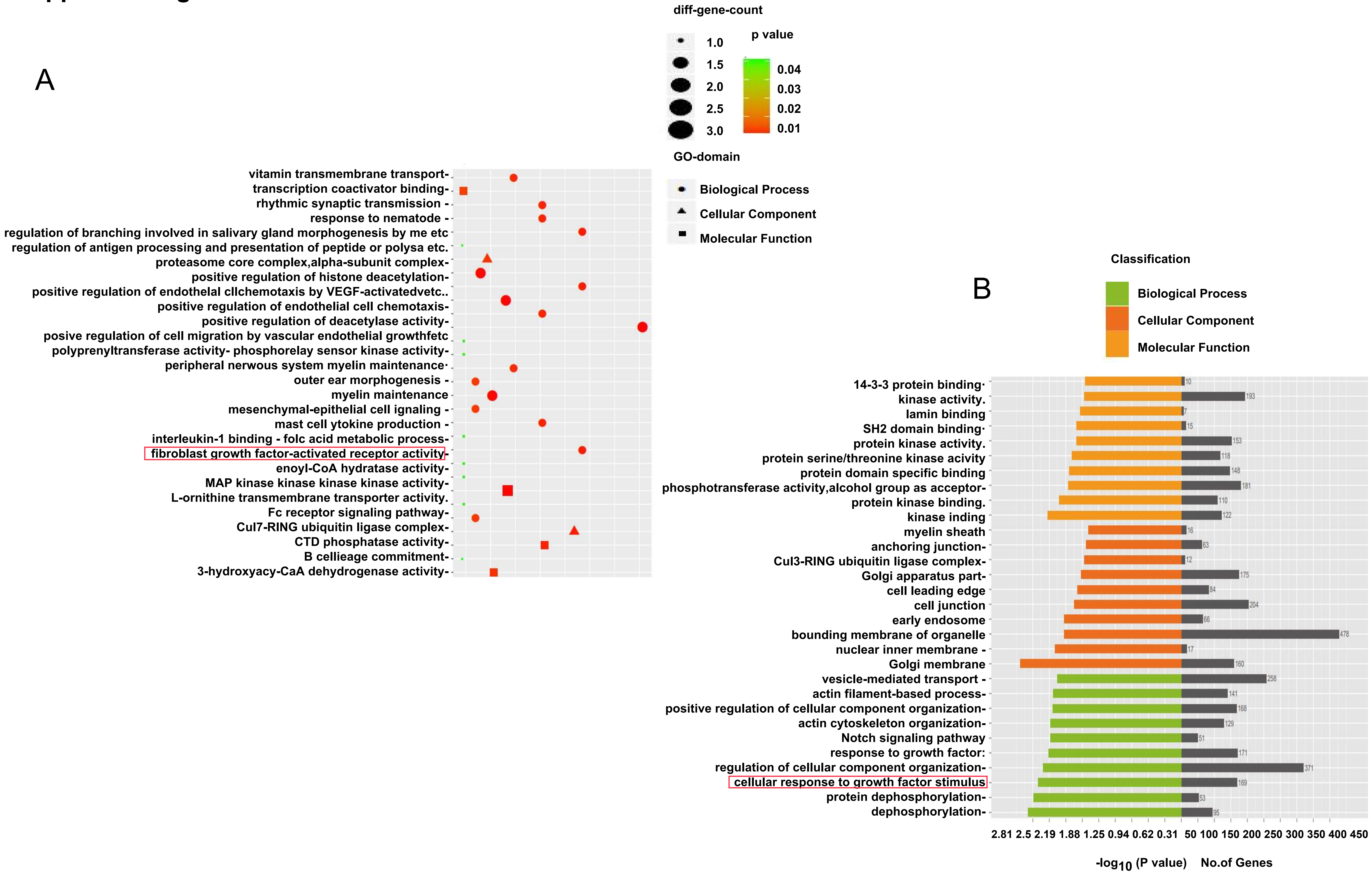

Bioinformatics analysis on the relationship between miR-210-3p and fibroblast activity. (A, B) GO analysis of the differentially expressed exosomal miR-210-3p derived from atrial myocytes showed enrichment in the regulation of proteins involved in fibroblast growth factor-activated receptor activity.

Supplement figure 4

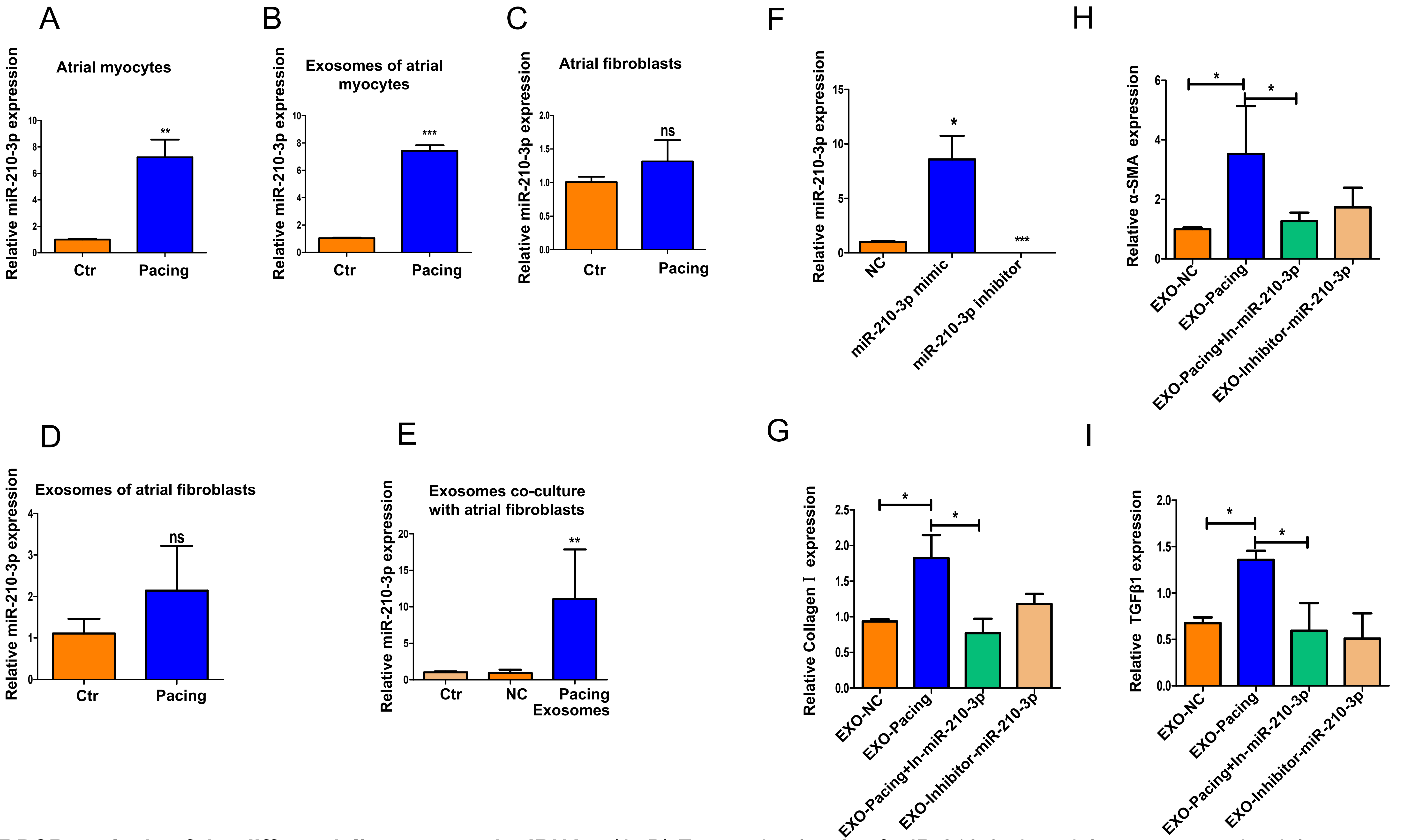

qRT-PCR analysis of the differentially expressed miRNAs. (A, B) Expression levels of miR-210-3p in atrial myocytes and atrial myocyte-derived exosomes. (C, D) Expression levels of miR-210-3p in atrial fibroblasts and atrial fibroblast-derived exosomes. (E) Coculture of atrial fibroblasts with exosomes derived from atrial myocytes (***P<0.001 compared with the Ctr and NC groups). (F) qRT–PCR analysis of the transfection efficiency of atrial myocytes (*P<0.05 and ***P<0.001 compared with the Ctr group). (G-I) The mRNA levels of α-SMA, collagen Ⅰ and TGFβ1 were evaluated using qRT–PCR (*P<0.05).

Supplement figure 5

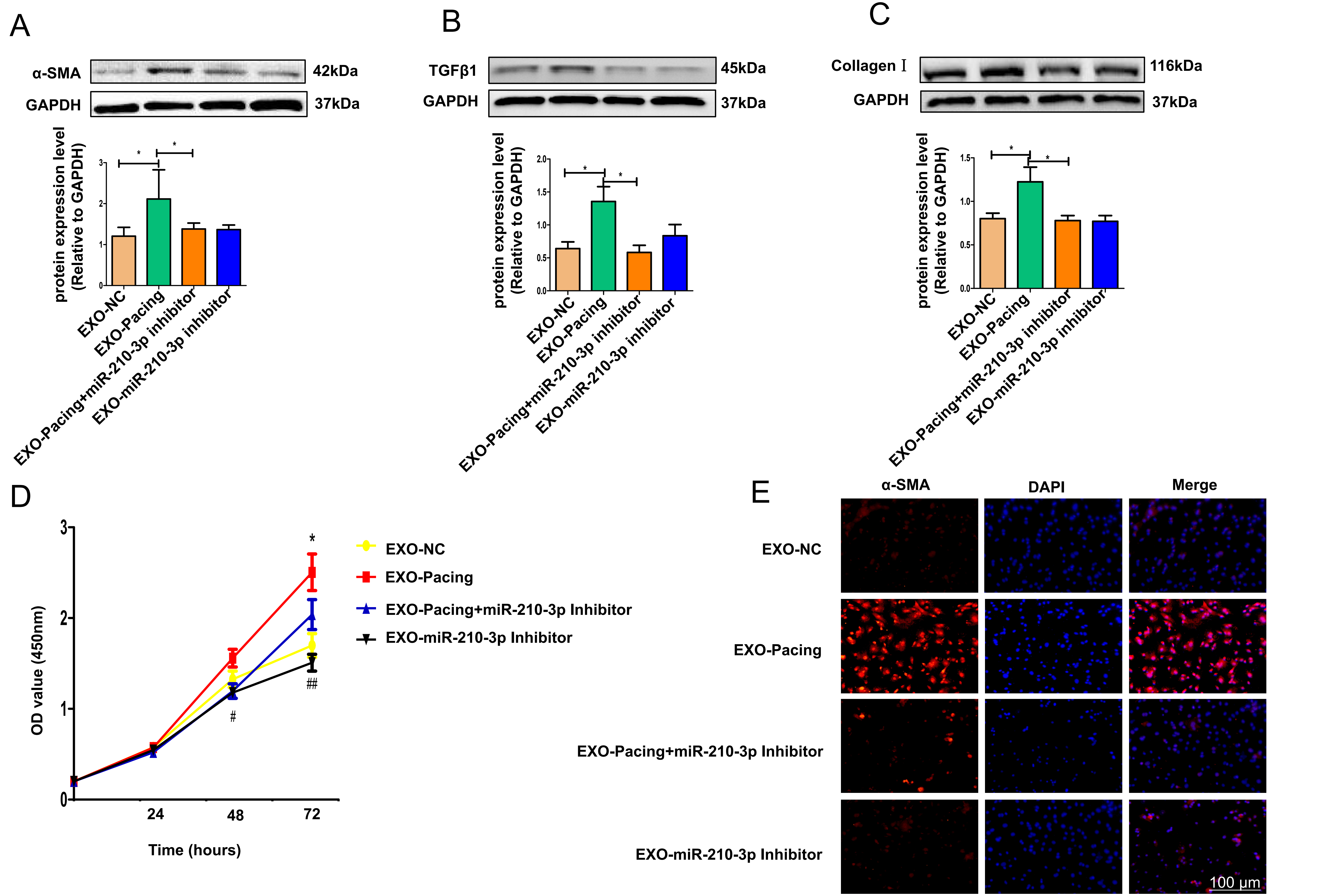

Exosomal miR-210-3p derived from atrial myocytes promotes atrial fibroblast proliferation and activation. (A-C) Western blot analysis of protein levels in atrial fibroblasts treated with atrial myocyte-derived exosomes (*P<0.05). (D) CCK-8 assay to detect the proliferation of atrial fibroblasts treated with atrial myocyte-derived exosomes (*P<0.05 compared with EXO-NC, and #P<0.05 and ##P<0.01 compared with EXO-Pacing). (E) Immunofluorescence staining showing the relative expression levels of α-SMA (red) and DAPI (blue) in atrial fibroblasts. Scale bar: 100μm.

Supplement figure 6

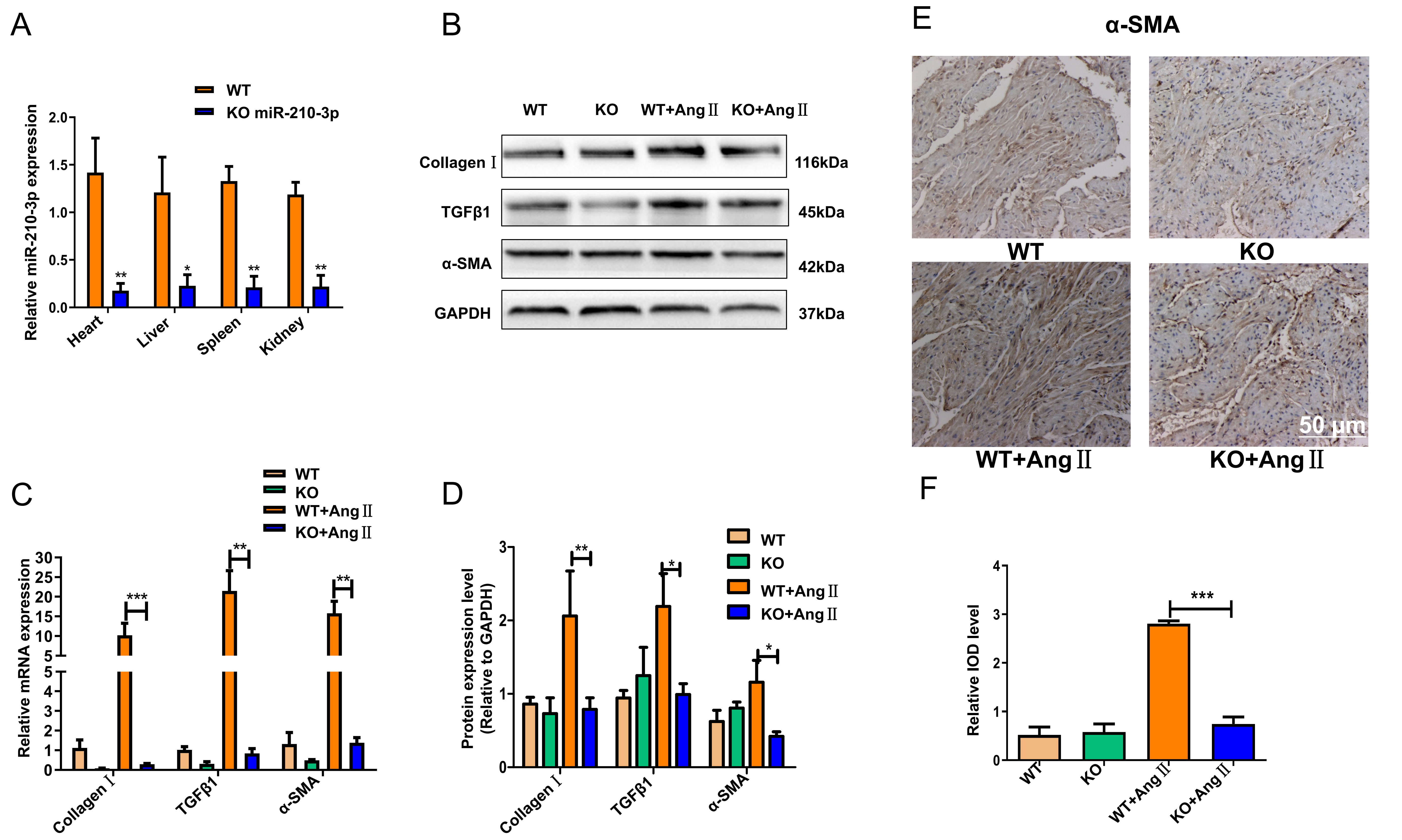

Effects of miR-210-3p KO on atrial fibrosis in rats. (A) qRT–PCR analysis of the expression of miR-210-3p in various organs of rats (n=7; *P<0.05 and **P<0.01 compared with the WT group). (B, D) Western blot analysis of TGFβ1, α-SMA and Collagen Ⅰ expression levels in atrial tissues (n=7; *P<0.05 and **P<0.01 compared with the KO+Ang II group). (C) qRT–PCR analysis of TGFβ1, α-SMA and Collagen Ⅰ expression levels in atrial tissues (n=7; **P<0.01 and ***P<0.001 compared with the KO+AngⅡ group). (E, F) Immunohistochemical staining for α-SMA; scale bar: 50 μm (n=7; ***P<0.001 compared with the KO+AngⅡ group).

Supplement figure 7

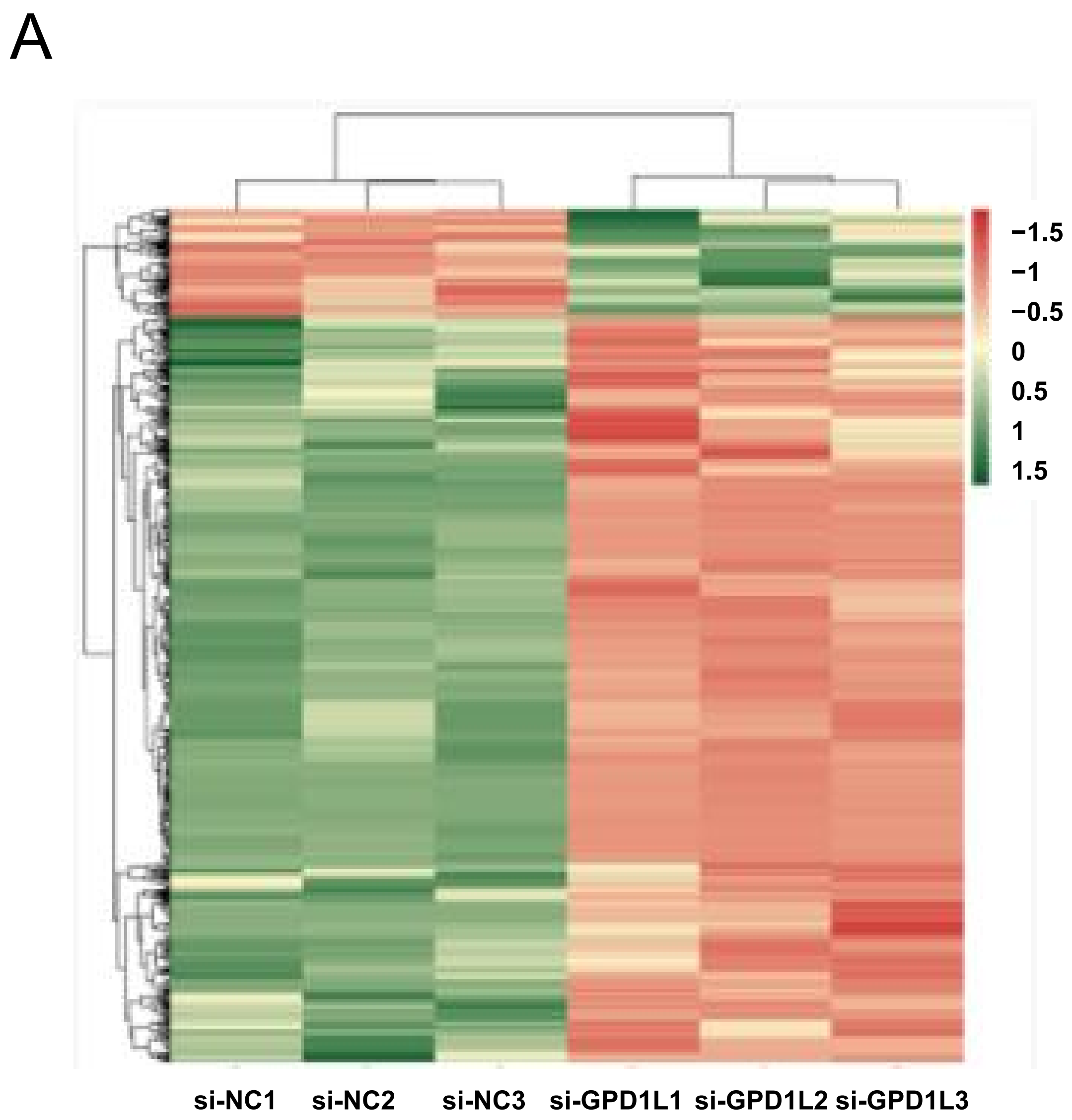

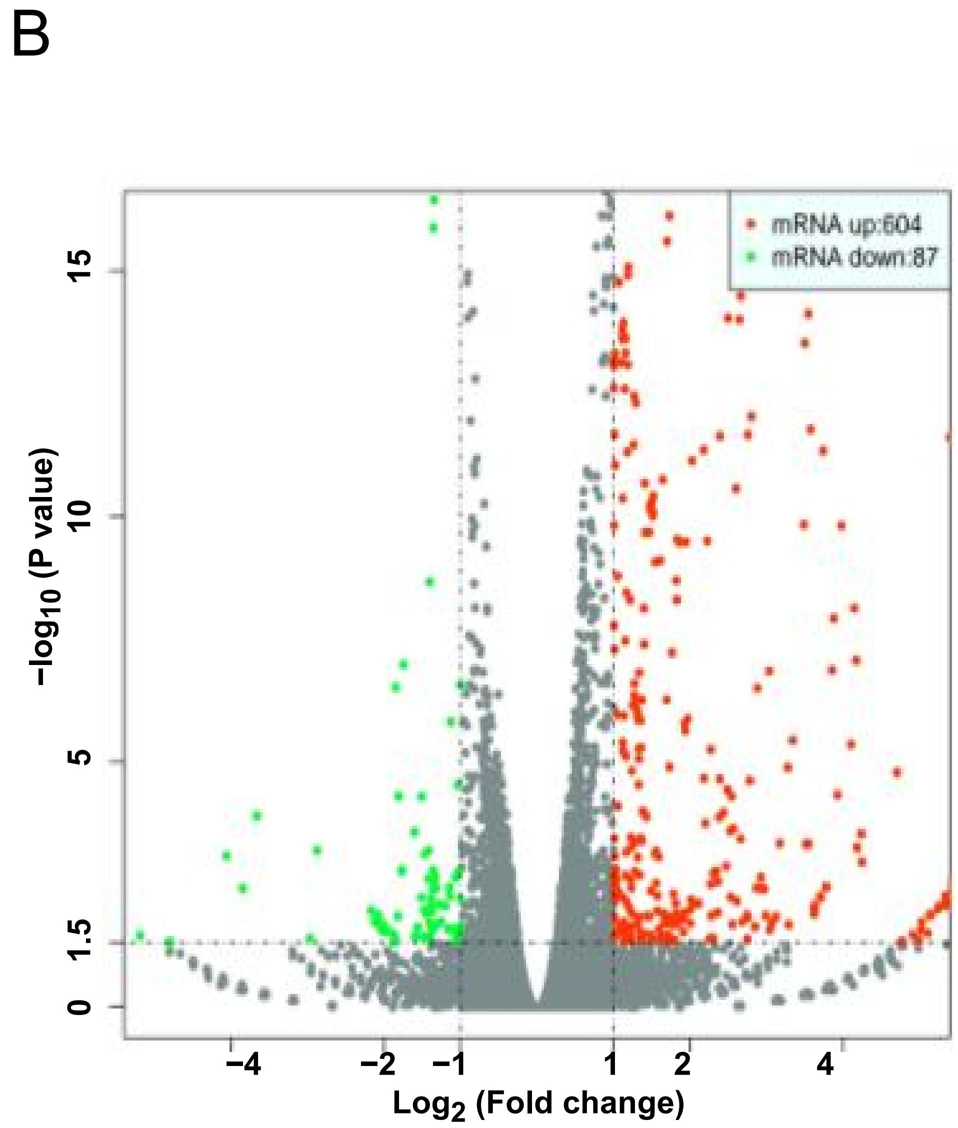

RNA sequencing analysis. (A, B) RNA-Seq analysis of differentially expressed mRNAs showing that GPD1L regulates atrial fibroblast proliferation and activation.

Supplement figure 8

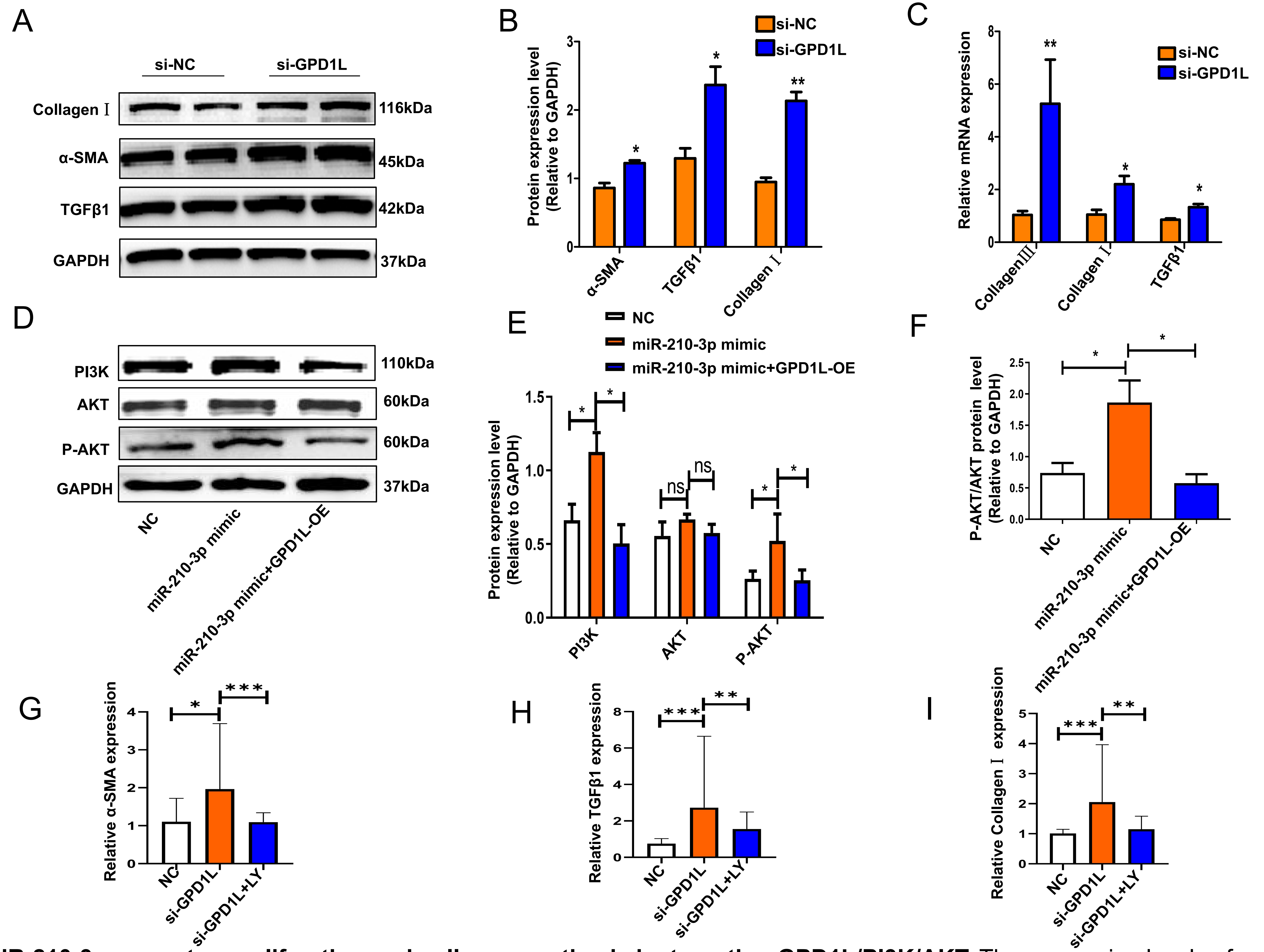

miR-210-3p promotes proliferation and collagen synthesis by targeting GPD1L/PI3K/AKT. The expression levels of proteins (A, B) and mRNAs (C) in the si-NC and si-GPD1L groups (*P<0.05 and **P<0.01). (D, E) Western blot analysis of PI3K, p-AKT and AKT levels in atrial fibroblasts (*P<0.05). (F) The ratio of p-AKT/AKT levels (*P<0.05). (G-I) qRT-PCR analysis of α-SMA, collagen I and TGFβ1 expression in atrial fibroblasts (*P<0.05, **P<0.01, and ***P<0.001).
